# Characteristics of interprofessional rehabilitation programs for patients with chronic low back pain evaluated in the literature: a scoping review protocol

**DOI:** 10.1186/s13643-023-02275-5

**Published:** 2023-06-29

**Authors:** Sintayehu Daba Wami, Solomon Fasika, Catherine Donnelly, Kassahun Alemu Gelaye, Abdul Pullatayil, Jordan Miller

**Affiliations:** 1 Department of Environmental and Occupational Health and Safety, Institute of Public Health, University of Gondar, Gondar, Ethiopia; 2 Department of Physiotherapy, School of Medicine, University of Gondar, Gondar, Ethiopia; 3 School of Rehabilitation Therapy, Queen’s University, Kingston, Canada; 4 Department of Epidemiology and Biostatistics, Institute of Public Health, University of Gondar, Gondar, Ethiopia; 5 Health Sciences Library, Queen’s University, Kingston, Canada

**Keywords:** Low back pain, Interdisciplinary, Interprofessional, Multidisciplinary, Rehabilitation

## Abstract

**Background:**

Interprofessional rehabilitation programs have demonstrated effectiveness at improving health-related quality of life, function, work abilities, and reducing pain, for patients with chronic low back pain (CLBP). However, the characteristics of interprofessional rehabilitation programs vary widely across studies. Therefore, clarifying and describing key characteristics of interprofessional rehabilitation programs for patients with CLBP will be valuable for future intervention design and implementation. This scoping review aims to identify and describe the key characteristics of interprofessional rehabilitation programs for patients with CLBP.

**Methods:**

Our scoping review will follow the framework developed by Arksey and O'Malley, further enhanced by Levac et al. and the Joanna Briggs Institute (JBI). Electronic databases, including MEDLINE, EMBASE, CINAHL, PsycINFO, SCOPUS, PubMed, Web of Science, and Cochrane Library, will be searched to identify relevant published studies. Our scoping review will consider all primary source peer-reviewed published articles that evaluated interprofessional rehabilitation programs for adults with CLBP from all countries and any therapeutic settings.

The Covidence software will be used to remove duplicates, article screening, record the step-by-step selection process, and data extraction. The analysis will involve a descriptive numerical summary and narrative analysis. Data will be presented in graphical and tabular format based on the nature of the data.

**Discussion:**

This scoping review is expected to provide a source of evidence for developing and implementing interprofessional rehabilitation programs in new settings or contexts. As such, this review will guide future research and provide key information to health professionals, researchers and policymakers interested in designing and implementing evidence and theory-informed interprofessional rehabilitation programs for patients with CLBP.

**Trial registration:**

https://osf.io/rquxv.

## Background

Low back pain (LBP) is a major public health problem globally [1, 2], incurring serious health system burdens and economic consequences on individuals, societies, and governments [3–5]. Chronic low back pain (CLBP) is defined as “a deep, aching, dull or burning pain or discomfort, localized below the costal margin and above the inferior gluteal folds, with or without pain referred into leg persisting for a period of greater than 12 weeks” [6, 7]. In addition to pain and functional limitations, people with CLBP often experience anxiety, depression, and poor health-related quality of life (HRQoL) at higher rates than those without low back pain [8].

CLBP is among the leading cause of years lived with disabilities (YLDs) worldwide [1, 14]. According to the Global Burden of Disease (GBD) 2017 estimates, the number of people with LBP increased from 377.5 million in 1990 to 577.0 million in 2017 [1, 9]. Hence, it remains a common problem in both high and low- and-middle-income countries [8, 10, 11]. The growing number of years lived with disability associated with LBP is being driven by increases in YLDs in low-and-middle-income countries [1, 9].

The complex, dynamic and multifactorial nature of CLBP makes it challenging to manage [12]. Increasingly, CLBP is well explained within a biopsychosocial model [13]. Interprofessional rehabilitation is recommended to address the multiple biological, psychological, and social factors associated with CLBP [14–16]. Recent studies indicate that an interprofessional rehabilitation program is effective in improving health-related quality of life, reducing pain, and improving the function and work participation of patients with CLBP [14–16]. However, currently, there is no single well-accepted description of an interprofessional rehabilitation program, and it is unclear what the optimal components of the interventions are, and which healthcare professionals shall be involved [6, 17]. Often, interprofessional rehabilitation programs have been defined as interventions consisting of at least two or more combinations of physical, psychological, social, vocational, and behavioral components delivered by a team of professionals with different backgrounds [15, 18]. The elements, duration, intensity, professionals involved, and descriptions of interprofessional rehabilitation programs vary widely across studies [18, 19], making it challenging to implement the evidence in new settings, especially in low- and middle-income countries. There is no evidence that an intervention component, duration, or setting of interprofessional rehabilitation programs described in the evaluated studies is superior to any of the other study treatment plan [20]. In addition, the health outcomes assessed were also heterogeneous, and there is no agreement on the measurement instrument [19].

Furthermore, heterogeneity in the contents, intensity of the interventions, and choice of outcomes may hamper comparisons between studies, knowledge synthesis, and conclusiveness of the evidence. To our knowledge, there is no published scoping review on descriptions and components of interprofessional rehabilitation programs for patients with CLBP. A synthesis of the key characteristics of interprofessional rehabilitation programs may facilitate the design and implementation of future interprofessional rehabilitation programs [21]. Hence, this scoping review aims to synthesize the descriptions and characteristics of interprofessional rehabilitation programs for people with CLBP evaluated in the literature.

### The rationale for a scoping review

A scoping review is selected as an appropriate and rigorous evidence synthesis approach for reviewing a heterogeneous body of evidence and mapping rapidly the key concepts underpinning a research area [22–24]. A preliminary search of the CINAHL (EBSCO), MEDLINE (Ovid), SCOPUS, Cochrane Library, and JBI Evidence Synthesis was performed in June 2022, and no current or underway scoping reviews on the topic were identified.

The evidence on the effectiveness of interprofessional rehabilitation programs for CLBP has been synthesized in systematic reviews of randomized controlled trials [25, 26]. However, the characteristics of interprofessional rehabilitation programs varied substantially across studies [17], and the systematic reviews have not synthesized the characteristics of each of the interprofessional rehabilitation programs evaluated. Hence, implementing the evidence from these systematic reviews in new settings is challenging. Further, systematic reviews of effectiveness have been limited to randomized control trials and have not included interprofessional rehabilitation programs for CLBP that have been evaluated through other study designs. Characteristics of the interprofessional rehabilitation programs that have been tailored to particular settings or contexts may be particularly beneficial for developing and evaluating interprofessional rehabilitation programs in new settings, including those with resource limitations.

This scoping review aims to provide a fulsome description of characteristics of interprofessional rehabilitation programs for CLBP evaluated in the peer-reviewed literature to support the development of interprofessional rehabilitation programs in new settings or contexts without limiting the search to particular trial designs. We anticipate the scoping review findings will provide a comprehensive overview of the characteristics of interprofessional rehabilitation programs, including professions involved, program components or interventions, frequency, duration, and theoretical foundations of interprofessional rehabilitation programs. 

## Methods

### Protocol design

Our scoping review will follow the framework developed by Arksey and O'Malley [22], which has been further enhanced by Levac et al. [27] and the Joanna Briggs Institute (JBI) [24]. The review process will include the following six stages: (1) identifying the research question; (2) identifying relevant studies; (3) selecting studies using an iterative team approach; (4) charting the data using the data extraction framework; (5) collate, summarize and report the results; and (6) consultation with key stakeholders [22, 27].

Our research team includes researchers from multiple disciplines, including team members with expertise in low back pain, interprofessional rehabilitation, and knowledge syntheses.

### Stage 1: identifying the research questions

Through discussion with the research team, the overarching research question guiding this review is: what are the key characteristics of interprofessional rehabilitation programs for patients with CLBP that have been evaluated in the peer-reviewed literature?

More specifically, this scoping review will try to address the following research questions:

1. What types of interventions have been included in interprofessional rehabilitation programs used to treat patients with CLBP?
2. How are interprofessional rehabilitation programs for patients with CLBP described across the studies?
3. What is the duration and intensity/frequency of visits included in interprofessional rehabilitation programs for patients with CLBP?
4. What health professionals have been involved in interprofessional rehabilitation programs for patients with CLBP?
5. What health outcomes have been assessed in interprofessional rehabilitation programs for patients with CLBP?
6. In what setting have interprofessional rehabilitation programs for patients with CLBP been provided?

### Stage 2: identifying relevant studies

#### Eligibility criteria

The JBI updated guidance for scoping review [24] recommends including detailed inclusion criteria in the protocol, containing the PCC (Population, Concept, and Context) elements. Accordingly, we have developed the following inclusion and exclusion criteria to guide the search and help identify relevant studies (Table 1).

**Table 1:** Inclusion criteria for this scoping study

Criteria	Descriptions
*Population*	Our scoping review will consider studies that included adults (18 and above years of age) experiencing CLBP. CLBP is defined as pain localized below the costal margin and above the inferior gluteal folds, with or without leg pain, that persists for three months or more to generally accepted scientific criteria. Studies that evaluate interprofessional rehabilitation programs for multiple pain conditions will be included only if at least 50% of participants are reported to have CLBP. We included adults because they are the most at-risk population, and the available evidence indicates that 75% of people with CLBP are adults [12] and nearly 70% of years lost due to disability were among the working-age population (20–65 years) [28]
*Concept*	Our scoping review will include any primary source research studies that evaluated multidisciplinary or interdisciplinary or interprofessional or team-based rehabilitation programs, consisting of a combination of two or more physical, psychosocial, social, vocational, and behavioral components delivered by a team of two or more health professionals with different backgrounds. Outcomes reported in the study could include health outcomes (e.g., physical functioning, pain intensity, HRQoL, Workability), cost outcomes (e.g. cost-utility, cost-effectiveness), or experiential outcomes of patients or providers (e.g. satisfaction, acceptability) [29]
*Context*	We will consider evidence from all countries and therapeutic settings (e.g., primary healthcare, inpatient, pain clinics, rehabilitation centers, and outpatient settings)

#### Types of sources

All primary published studies with any methodological approach (quantitative, qualitative, and mixed design research) will be the sources of evidence considered in this scoping study. The scoping review will include all studies that evaluate interprofessional rehabilitation programs for CLBP. The reference lists of systematic reviews and relevant studies identified through our search will be scanned to identify relevant primary research articles. However, we will not consider grey literature due to the time constraints we have in searching a large volume of grey literature, the lack of resources to manage a large volume of information found, and the inability to cover all grey literature repositories. As it is known, grey literature is less formally archived evidence, and searching will be time-consuming and inefficient. In addition, we wanted to include peer-reviewed high-quality evidence with a robust methodology and low risk of bias. In addition, opinion papers, all types of systematic reviews, and undergrad students’ theses not published in peer-reviewed journals will be excluded. In addition, commentaries, book reviews, and studies focusing on single-model interventions will also be excluded.

#### Search strategy

Electronic databases like MEDLINE, EMBASE, CINAHL, PsycINFO, SCOPUS, and Web of Science will be searched to identify relevant published studies.

A systematic approach will be followed to search for relevant articles using a combination of medical subject heading (MeSH) and keywords related to an interprofessional rehabilitation program and CLBP. A Queen’s University librarian (AP) helps to refine the search strategy, keywords, and MeSH terms for each database. In addition, titles of articles from an initial search will also be checked to refine search terms. The following initial key search terms are developed by the research team and refined with the support of Queen’s University librarian (AP):

##### Key concepts

- Concept 1: Chronic low back pain, Low Back Pain [MeSH Term], Low back ache, Back pain, Low backache, Lumbago, Mechanical low back pain, Lumbar pain
- Concept 2: Multidisciplinary, Interdisciplinary, Interprofessional, Team, Transdisciplinary, Team-based, Collaborative, Multi-professional
- Concept 3: Rehabilitation, Treatment, Management, Intervention, Approach, Care, Therapy

A comprehensive search strategy will be developed using Boolean operators and truncation to fit each database. First, each term identified under each concept will be entered into different databases to identify subject headings and similar keywords. Then, we will search one concept at a time, and terms within each concept will be connected with ‘OR’ Boolean operator. Finally, we will search all identified MeSH terms and keywords together in different databases. We will use the Boolean operator ‘AND’ between each concept. An iterative process will be applied to identify and develop a list of key search terms. The librarian (AP) in our research team will test relevant MeSH terms, keywords, and filters to enhance the sensitivity and specificity within each database. A sample search strategy for OVID MEDLINE and CINAHL databases is found in Appendix 1. The search strategy we use in different bibliographic databases will be available upon request from the authors.

The search will be limited to articles published in English due to the time and costs associated with translating articles published in other languages. However, there will be no date limits applied to our search.

### Stage 3: study selection

The reference lists and abstracts of articles found from each database will be retrieved and imported into Covidence software [30]. The Covidence software will be used to remove duplicates and save references for further screening and data extraction process. Covidence was chosen to remove duplicates due to its accuracy, specificity and sensitivity to detect de-duplicates [31].

The study selection process will be conducted independently by two reviewers. Disagreement will be resolved by mutual consensus or by a third reviewer through a two-step process using Covidence software to record the step-by-step selection process according to our inclusion criteria. First, the two reviewers will independently review all the titles and abstracts of articles against the inclusion and exclusion criteria. As Levac et al. [27] suggested, the two reviewers will also meet part-way through the abstract review process to discuss any difficulties or confusion related to study selection and refine the search strategy. Hence, we will follow an iterative process and check whether the inclusion/exclusion criteria are followed consistently throughout the review process. Full-text articles will be reviewed when reviewers agree, based on the abstract, that the article is likely to be included and when it is difficult to decide based on the title and abstract. Next, the two reviewers will independently review the full articles of each selected for full-text review. We will compute Cohen’s kappa statistic to measure the inter-rater reliability (level of agreement) between the two reviewers [32]. A third reviewer will be consulted to decide the final inclusion when a disagreement happens between the two reviewers at either of the two stages of the screening process. Each reviewer will note the reasons when excluding the articles.

A flow chart will be prepared to show the study selection procedure at each stage of the review, including the reasons for exclusion. The study selection process will be reported using Preferred Reporting Items for Systematic Reviews and Meta-Analyses for Scoping Reviews (PRISMA-ScR) flow diagram [33]. Since a scoping review methodology does not indicate the necessity for evaluating study quality and risk of bias assessment criteria [22, 24], we include all articles that fulfill the inclusion criteria. Hence, we will not perform a critical appraisal of included studies.

### Stage 4: charting the data

Two reviewers will independently extract the data from articles included in the scoping review using the data charting framework in the software program, Covidence. A draft extraction form is provided (see Table 2). We will regularly update and refine the data charting framework and consider it an iterative process [24]. Any uncertainties related to the data extraction will be resolved through discussion between the research team. Moreover, regular discussions will be held between the research team to refine the framework.

**Table 2:** Data charting framework

S.no	Category/variable	Description
1	Authors	List the name of the authors of the study
2	Study title	Write the full title of the study/source
3	Sources/journal	Specify the name of the journal/publisher
4	Publication type	Specify sources of the evidence, e.g., published articles, unpublished studies, reports, conference proceedings
5	Year of publication	Specify the year the study was published or available
6	Objective(s) of the study	Write the stated objectives of the study
7	Study design	Describe the methodological approach followed (e.g., RCT, Observational studies, qualitative studies, etc.)
8	Country • Location of the study • Economic status of the country	Specify the location where the study was conducted Describe the economic status/income levels of the country based on the World Bank classification (e.g., High income, low and middle income, low income, lower middle income, middle income, upper middle income)
9	Descriptions of the study population/targeted population • Sociodemographic Characteristics ◦ Age group ◦ Sex • Operational definition of CLBP	Describe the characteristics of the targeted population (e.g., sociodemographic characteristics, health condition) Specify the definition of CLBP used in the study, and the inclusion criteria applied
10	Descriptions of characteristics of the interprofessional rehabilitation programs	
	**•** Detail description of the intervention (e.g., Components of the intervention included)**•** Definitions	◦ Describe the type(s) of the interventions evaluated in the study ◦ Describe the contents of the interventions ◦ Specify the definition used to describe the intervention
	• Delivery mode	Describe how the intervention is delivered
	• Duration and frequency of the intervention	State for how long the intervention is delivered and its frequency
	• Length of Follow up	Describe for how long the patients followed after the intervention
	• Health professionals involved	Describe by whom the intervention was delivered
	• The setting of the intervention	Describe the context/setting of the intervention (e.g., inpatient, primary care, outpatient, pain clinic, rehabilitation center)
	• Theoretical foundations	Describe the underpinning theory of the intervention, the theoretical foundation of the rehabilitation programs/ theoretical basis of interprofessional rehabilitation programs (e.g., biopsychosocial model, Cognitive behavioural model, fear avoidance model)
11	Reported outcomes and experiences assessed and measurement approaches used	Describe the intervention outcomes reported in the study, including health outcomes (e.g., physical functioning, pain intensity, HRQoL, Workability), cost outcomes (e.g., cost-utility, cost-effectiveness), or experiential outcomes (e.g. satisfaction, acceptability)
12	Barriers and facilitators	Describe barriers and facilitators for the implementation of the intervention

Before the formal data extraction process, two reviewers will test the framework independently by extracting data from 10% of the included studies to ensure that their data extraction approach is uniform, and the framework is consistently used (reliability between extractors). We will only begin formal extraction when the percentage agreement is above 90% between the two reviewers. Inconsistency in extracted data between the two reviewers will be discussed until consensus is reached or resolved by a third reviewer [27].

Data to be extracted from relevant selected studies will include information about the author, year of publication, the objective of the study, study design, detailed descriptions of the interventions, country, settings, targeted population, and types of outcomes assessed (Table 2).

### Stage 5: collating, summarizing, and reporting the results

The analysis will involve a descriptive numerical summary (e.g., frequency, percentage) and a narrative summary. First, we will apply a numerical description of the characteristics of included studies, including the overall number of studies included, years of publication, types of study designs, countries where studies were conducted, characteristics of the study population, detailed description of interventions, health professions involved in delivering the program, delivery mode, frequency and duration of the program, and settings of the intervention (Table 2).

The results will be reported through a thematic presentation and tables that align with the purpose of the scoping study. In addition, data will be presented in graphical representations such as pie charts and bar graphs when appropriate. Finally, we will discuss the meaning of the findings and implications for future research, policy, and clinical practice [27].

In this analysis, we will compare and describe interprofessional rehabilitation programs for people with CLBP evaluated across studies. The analysis will also explore the types of outcomes that have been reported in interprofessional rehabilitation programs for people with CLBP. In addition, we will try to show gaps in the descriptions and contents of an interprofessional rehabilitation program and indicate areas where further analysis is required. This scoping review will adhere to the Preferred Reporting Items in Systematic Reviews and Meta-analyses extension for scoping reviews (PRISMA-ScR) [33]. Any deviation from this protocol will be clearly detailed and justified in the full review report. The findings at this stage will be used to inform the stakeholders’ consultation process.

### Stage 6: consultation with key stakeholders

Although Arksey and O'Malley (Arksey and O'Malley, 2005) suggest that consultation is an optional stage in conducting a scoping review, Levac et al. (Levac et al. 2010) argue that it should be a required component to add methodological rigour. Accordingly, we will conduct consultation with stakeholders to share preliminary results, to help identify potential meaning from the findings, obtain their feedback about the findings not revealed by the scoping study, and identify additional references about potentially relevant studies which offer extra value to a scoping study. Hence, we will use a preliminary finding to inform the consultation, which enables stakeholders to build on the evidence and offer a higher level of meaning and perspective to the preliminary findings.

Experts in CLBP, including healthcare providers and researchers in CLBP and interprofessional rehabilitation, will be consulted, thus offering important insights beyond what has been reported from the available evidence. We will conduct a focus group discussion (FGD) through a Zoom meeting with six to eight stakeholders. We will purposively recruit participants based on their expertise and lived experiences and send an invitation with a consent form through email. We will ask for any additional insights on how to interpret the findings of what has been reported in the literature and identify important gaps for future research. Focus groups will be video/audio-recorded, transcribed verbatim, and analyzed using a descriptive qualitative approach.

## Discussion

This review aims to synthesize the characteristics of interprofessional rehabilitation programs for patients with CLBP evaluated in the literature. The characteristics described will include professions involved, program components or interventions, frequency, duration, the outcome evaluated, and theoretical foundations of interprofessional rehabilitation programs for patients with CLBP evaluated in the literature to date. Synthesizing and describing the characteristics and components of interprofessional rehabilitation programs is expected to provide valuable evidence to inform new program development and implementation. For example, this review will constitute the initial step in a multistage research project to identify and develop an evidence and theory-informed interprofessional rehabilitation program for patients with CLBP that can be implemented in Ethiopia. This scoping review is expected to provide evidence for this, and other initiatives aimed at developing and implementing interprofessional rehabilitation programs in new settings or contexts. As such, this review will be beneficial in guiding future research and providing key information to health professionals, researchers and policymakers interested in designing and implementing evidence and theory-informed interprofessional rehabilitation programs for patients with CLBP.

## Figures and Tables

**Table:**
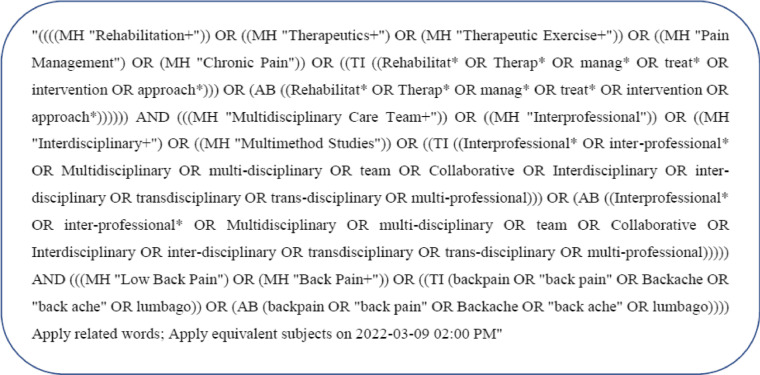
Box 1. Sample of search terms from the CINAHL database

## Data Availability

Not applicable.

## Appendix 1: Search strategy

Database: Ovid MEDLINE(R) ALL < 1946 to June 04, 2022 >

**Table:**

#	Query	Results from 4 June 2022
1	exp Rehabilitation/	334,918
2	exp Therapeutics/	4,937,770
3	exp Pain Management/	38,802
4	(Rehabilitat* or Therap* or manag* or treat* or intervention or approach* or care).mp. [mp = title, abstract, original title, name of substance word, subject heading word, floating sub-heading word, keyword heading word, organism supplementary concept word, protocol supplementary concept word, rare disease supplementary concept word, unique identifier, synonyms]	12,195,314
5	1 or 2 or 3 or 4	13,714,858
6	exp Patient Care Team/	71,854
7	exp Interdisciplinary Studies/	1,163
8	(Interprofessional* or inter-professional* or Multidisciplinary or multidisciplinary or team or Interdisciplinary or inter-disciplinary or transdisciplinary or trans-disciplinary or collaborative or multi-professional).mp. [mp = title, abstract, original title, name of substance word, subject heading word, floating sub-heading word, keyword heading word, organism supplementary concept word, protocol supplementary concept word, rare disease supplementary concept word, unique identifier, synonyms]	419,846
9	6 or 7 or 8	419,859
10	exp back pain/or exp low back pain/	42,434
11	(backpain or "back pain" or Backache or "back ache" or lumbago).mp. [mp = title, abstract, original title, name of substance word, subject heading word, floating sub-heading word, keyword heading word, organism supplementary concept word, protocol supplementary concept word, rare disease supplementary concept word, unique identifier, synonyms]	70,680
12	10 or 11	70,916
13	5 and 9 and 12	1,897

## Abbreviations

BMI: Body mass index
CINAHL: Cumulative Index to Nursing and Allied Health Literature
CLBP: Chronic low back pain
EMBASE: Excerpta Medica Database
GBD: Global Burden of Disease
HRQoL: Health-related quality of life
LBP: Low back pain
MEDLINE: Medical Literature Analysis and Retrieval System Online
MeSH: Medical Subject Heading
PRISMA-ScR: Preferred Reporting Items for Systematic Reviews and Meta-Analyses for Scoping Reviews
RCT: Randomized control trial
YLDs: Years lived with disabilities
